# Introducing the ‘Lived Experience’ section of the *South African Journal of Psychiatry*

**DOI:** 10.4102/sajpsychiatry.v30i0.2268

**Published:** 2024-04-30

**Authors:** Laila Asmal, Mehita Iqani

**Affiliations:** 1 Department of Psychiatry, Faculty of Medicine and Health Sciences, Stellenbosch University, Cape Town, South Africa; 2 South African Research Chair in Science Communication, Faculty of Arts and Social Sciences, Stellenbosch University, Stellenbosch, South Africa

The *South African Journal of Psychiatry (SAJP)* is introducing a ‘Lived Experience’ section, starting with this issue, aimed at enriching understandings of mental health. This initiative recognises that individuals facing, living with and recovering from mental illness bring unique expertise to the research project. As well as being the recipients of psychiatric research and beneficiaries of healthcare, they are important contributors of experiential knowledge that should be central to how the discipline understands, researches and treats mental health and recovery.

‘Lived Experience’ accounts are stories written by people who have first-hand experience of mental illness. These narratives offer insight into the real-life context of a person’s illness and how it affects their life. The inaugural contribution to this section comes from a person with lived experience of schizophrenia. It is a deeply personal narrative that sheds light on the complexities of this condition. Each ‘Lived Experience’ will be a unique narrative, and could reveal varied aspects of the mental health journey, including struggles, hopes and paths to recovery. They also offer the potential to play a vital role in broadening understanding and empathy in society more broadly, of how the human condition can be affected by mental illness.

One of the most important theorists of decolonisation and decoloniality was Frantz Fanon who, critically for the purposes of this project, also happened to be a black African psychiatrist.^1,2^ Decolonisation refers to the political process of achieving independence, whereas decoloniality refers to the ongoing project of recovering from, and undoing, the cultural, political, economic and mental legacies of colonialism.^3^ A decolonial theoretical framework is central to a project of publishing Lived Experiences of mental illness. Along with the move to ‘decolonise’ various fields of higher education pedagogy and research, there have been calls to do the same in the field of psychiatry. Many knowledge disciplines were bound up in the colonial project, but some might argue that psychiatry played a special role in ‘colonial characterisation of non-Western societies, their cultures and indigenous healing systems, as inferior’.^4^ Significant collective work is underway in the field to decolonise the knowledge systems that have come to define it.^5,6^

The ‘Lived Experience’ section aims to create a new space for experts in psychiatry to actively listen to the people afflicted by mental illness. It invites the inclusion in this scholarly journal of more diverse perspectives and narratives many of which have long been overshadowed by historical and systemic biases in understanding and treating mental illness, especially in marginalised communities and cultures. What might the scholarly community of psychiatry researchers learn from, for example, the story of a migrant from a war-ravaged country who has faced severe anxiety and depression? Or from an individual who is an involuntary mental healthcare user, and is navigating the challenges of an under-resourced hospital, uncertain about what awaits them and struggling to be understood because of language barriers? What are the specific daily hurdles faced by someone managing social anxiety disorder? What does the long journey of recovery in ‘stable patients’ look and feel like, for them?

Such narratives have the potential to offer vital insights into the complexities of mental health. They deepen and thicken the scholarly dialogues taking place within the pages of this journal.

Arguably, a key component of the decolonial project in psychiatry is to create new opportunities to listen to the people who are most impacted by the diseases studied and treated by psychiatrists. A linear model of science, and indeed science communication, frames highly qualified medical experts as the holders of knowledge, and ordinary people (especially those who have lacked social, economic and educational opportunities to advance materially) as the recipients of their expertise. But it bears asking how any expert knowledge about a condition can be constructed without the presence, and communication, of the experiences of those living with it.

In this spirit, it is necessary to create distance from the well-intentioned yet problematic idea of ‘giving voice to the voiceless’. As Gayatri Chakravorty Spivak has argued, people who are structurally marginalised by economic, political, cultural and scholarly power are not voiceless. The question is whether those in power are willing to listen.^7,8,9^ The voices and narratives of people with lived experience (PWLE) of mental illnesses have often been obscured by stigma and exclusion or dominated by the framings and translations of medical professionals who report on their lives in aggregated findings published in peer-reviewed journals. The Lived Experiences section in this journal seeks to amplify their voices without supplanting them or speaking for them. It challenges the notion that psychiatrists are the sole experts and benevolent guardians of knowledge about mental illness.

While psychiatrists play a significant role in shaping diagnostic and treatment pathways, it is essential to acknowledge the complex history tied to colonisation and its impacts, and to forge new ways to undo damaging aspects of that legacy. Western psychiatry’s predominance, imposed on different cultures, is a testament to this legacy. Members of the scientific faculty have a duty to recognise psychiatry’s role in marginalising the voices of PWLE. This initiative invites researchers to listen with humility, openness to learning and without judgement. Indeed, within African contexts, traditional healers have been acknowledged as promoters of social psychiatry because of their skills in forging empathy with their patients through attentive listening.^5^

The ‘Lived Experience’ section is a stride towards inclusivity and an invitation to create scholarly environments where PWLE narratives are embraced without intermediaries. This shift calls for a nuanced understanding of the roles of psychiatrists, recognising the lived experiences of those most affected by mental health challenges.

## Scope

The SAJP welcomes contributions from people experiencing mental illness currently, who have experienced it in the past, or who have cared for afflicted family members. Clinicians are encouraged to support patients in submitting articles to SAJP’s new section, Lived Experience accounts.

## Author requirements

We invite contributions of 1500 words or less, with a focus on clear and concise writing that emphasises points important for mental health professionals to hear. We have partnered with the South African Research Chair in Science Communication to provide narrative assistance, ensuring that each testimony is shared responsibly and authentically.

## How to submit as a person with lived experience

We welcome your contribution and are ready to collaborate with you to ensure that the narrative accurately reflects your experience. You will have the final say on publication, and we are open to publishing accounts anonymously if preferred. For more information or to submit your work, please email A/Prof. Laila Asmal at assoc_editor@sajp.org.za. For more information regarding the submission process for Lived Experience articles authors are encouraged to visit the SAJP website and navigate to the authors tab where they will be able to access the submission guidelines.
